# Aberrant dopamine transporter and functional connectivity patterns in *LRRK2* and *GBA* mutation carriers

**DOI:** 10.1038/s41531-022-00285-z

**Published:** 2022-03-03

**Authors:** Amgad Droby, Moran Artzi, Hedva Lerman, R. Matthew Hutchison, Dafna Ben Bashat, Nurit Omer, Tanya Gurevich, Avi Orr-Urtreger, Batsheva Cohen, Jesse M. Cedarbaum, Einat Even Sapir, Nir Giladi, Anat Mirelman, Avner Thaler

**Affiliations:** 1 Movement Disorders Unit, Neurological Institute, Tel Aviv Medical Center, Tel Aviv, Israel; 2 Laboratory for Early Markers of Neurodegeneration, Neurological Institute, Tel Aviv Medical Center, Tel Aviv, Israel; 3 Sackler Faculty of Medicine, Tel Aviv University, Tel Aviv, Israel; 4 Sagol School of Neuroscience, Tel Aviv University, Tel Aviv, Israel; 5 Sagol Brain Institute, Tel Aviv Sourasky Medical Center, Tel Aviv, Israel; 6 Department of Nuclear Medicine, Tel Aviv Sourasky Medical Center, Tel Aviv, Israel; 7 Biogen Inc, Cambridge, MA USA; 8 Coeruleus Clinical Sciences LLC, Woodbridge, CT USA

**Keywords:** Parkinson's disease, Parkinson's disease

## Abstract

Non-manifesting carriers (NMCs) of Parkinson’s disease (PD)-related mutations such as *LRRK2* and *GBA* are at an increased risk for developing PD. Dopamine transporter (DaT)-spectral positron emission computed tomography is widely used for capturing functional nigrostriatal dopaminergic activity. However, it does not reflect other ongoing neuronal processes; especially in the prodromal stages of the disease. Resting-state fMRI (rs-fMRI) has been proposed as a mode for assessing functional alterations associated with PD, but its relation to dopaminergic deficiency remains unclear. We aimed to study the association between presynaptic striatal dopamine uptake and functional connectivity (FC) patterns among healthy first-degree relatives of PD patients with mutations in *LRRK2* and *GBA genes*. *N* = 85 healthy first-degree subjects were enrolled and genotyped. All participants underwent DaT and rs-fMRI scans, as well as a comprehensive clinical assessment battery. Between-group differences in FC within striatal regions were investigated and compared with striatal binding ratios (SBR). *N* = 26 *GBA-*NMCs, *N* = 25 *LRRK2-*NMCs, and *N* = 34 age-matched nonmanifesting noncarriers (NM-NCs) were included in each study group based on genetic status. While genetically-defined groups were similar across clinical measures, *LRRK2*-NMCs demonstrated lower SBR in the right putamen compared with NM-NCs, and higher right putamen FC compared to *GBA*-NMCs. In this group, higher striatal FC was associated with increased risk for PD. The observed differential SBR and FC patterns among *LRRK2*-NMCs and *GBA*-NMCs indicate that DaTscan and FC assessments might offer a more sensitive prediction of the risk for PD in the pre-clinical stages of the disease.

## Introduction

Dopamine transporter (DaT)-single-photon emission computed tomography (DaTscan) is a sensitive imaging biomarker for early disease detection in Parkinson’s disease (PD)^1^, quantifying a membrane protein expressed exclusively in dopaminergic neurons^2^. In PD, a dorso-posterior to a ventro-anterior gradient of striatal dopamine depletion occurs, with dopamine depletion in the premotor phase starting in the posterior putamen prior to changes in the caudate nucleus^2^. Decreased striatal DaT uptake was detected among individuals with idiopathic rapid-eye-movement sleep behavior disorder (RBD), highlighting its’ role in the pre-motor phase of PD^3^. Yet, while DaTscans are sensitive to striatal presynaptic DaT density, its spatio-temporal resolution remains rather limited^4,5^.

Resting-state functional MRI (rs-fMRI) enables the assessment of the inter-regional temporal correlations (functional connectivity [FC]) patterns reflecting spontaneous neural activity^6,7^. Previous rs-fMRI studies reported patterns of FC changes within cortical and subcortical functional networks of the brain in both PD patients and non-manifesting carriers (NMCs) of mutations in the *LRRK2* genes^8–14^.

Recent findings based on data from the Parkinson’s Progressive Marker Initiative (PPMI) study reported higher striatal binding ratios (SBR) in *GBA-* NMC compared with *LRRK2*- NMC and healthy controls^15^. Therefore, understanding DaT downregulation and functional compensation dynamics in the preclinical phases of the disease is of great interest^15,16^.

In this study, we aimed to characterize presynaptic dopamine activity in NMC of mutations in the *GBA* and *LRRK2* genes using DaTscans and to investigate its association with striatal FC patterns in these genotypic groups.

## Results

### Demographic and clinical characteristics of the study participants

Genetic phenotyping resulted in three study groups that consist of; *N* = 34 nonmanifesting noncarriers (NM-NC), *N* = 26 *GBA*-NMCs, and *N* = 25 *LRRK2*-NMCs. *N* = 74 (86%) were right-handed, *N* = 9 (10.5%) were left-handed, and *N* = 3 (3.5%) were ambidextrous. No significant differences were observed between the study groups in age, gender, H&Y, handedness, UPDRS-III, UPDRS-total, MOCA, BDI, SCOPA-AUT, RBDq, NMS scores, and UPSIT scores (*p* > 0.05; in all cases) (Table 1). Both NMC groups showed higher, yet non–significant, mean LR compared to NM-NCs.

**Table 1:** Demographic and clinical characteristics of the three study groups.

	NM-NC	GBA NM-MC	LRRK2 NM-MC	*p*-value
Age	53.55 ± 10.51	52.85 ± 8.81	50.52 ± 7.56	0.44^a^
Gender (F/M)	18/16	12/14	13/12	0.91^b^
Handedness				
⁃ Right/left/ambidextrous	29/4/2	25/1/0	20/4/1	0.19^c^
UPDRS-III	0.87 ± 1.26	0.61 ± 1.1	0.76 ± 2.05	0.86^a^
UPDRS- Total	6.62 ± 5.32	4.50 ± 2.8	5.96 ± 5	0.26^a^
MOCA	26.50 ± 2.68	26.69 ± 2.56	27.68 ± 2.68	0.28^a^
UPSIT	31.39 ± 6.95	30.35 ± 7.42	37.12 ± 9.11	0.66^a^
BDI	2.67 ± 6.21	5.28 ± 5.60	4.56 ± 4.77	0.08^a^
SCOPA -AUT	7.03 ± 6.21	6.52 ± 7.00	7.18 ± 6.22	0.93^a^
RBDq	2.00 ± 1.52	1.56 ± 2	1.43 ± 1.85	0.44^a^
NMS	3.18 ± 3.20	2.80 ± 3.24	2.95 ± 1.92	0.88^a^
LR	6.33 ± 17.00	11.38 ± 26.23	12.00 ± 26.00	0.57^a^
⁃ Low (<70) [*N*, %]	34, 97%	24, 92%	23, 92%	–
⁃ High (>70) [*N*,%]	1, 3%	2, 8%	2, 8%	–

Unless otherwise stated, all values are expressed as mean ± SD.

^a^1 × 3 ANOVA.

^b^*χ*^2^ test.

^c^Kruskal-Wallis test. *H&Y* Hohen & Yahr stage, *UPDRS* Unified Parkinson’s disease rating scale, *MOCA* Montreal cognitive assessment. *UPSIT* university of Pennsylvania smell identification test, *BDI* Beck’s depression inventory, *SCOPA-AUT* Scale of Outcome in Parkinson’s disease Autonomic Dysfunction questionnaire, *RBDq* REM sleep behavior disorder questionnaire, *NMS* nonmotor symptoms questionnaire, *LR* likelihood ratio.

### Between-groups differences in DaTscan SBRs

**Fig. 1:**
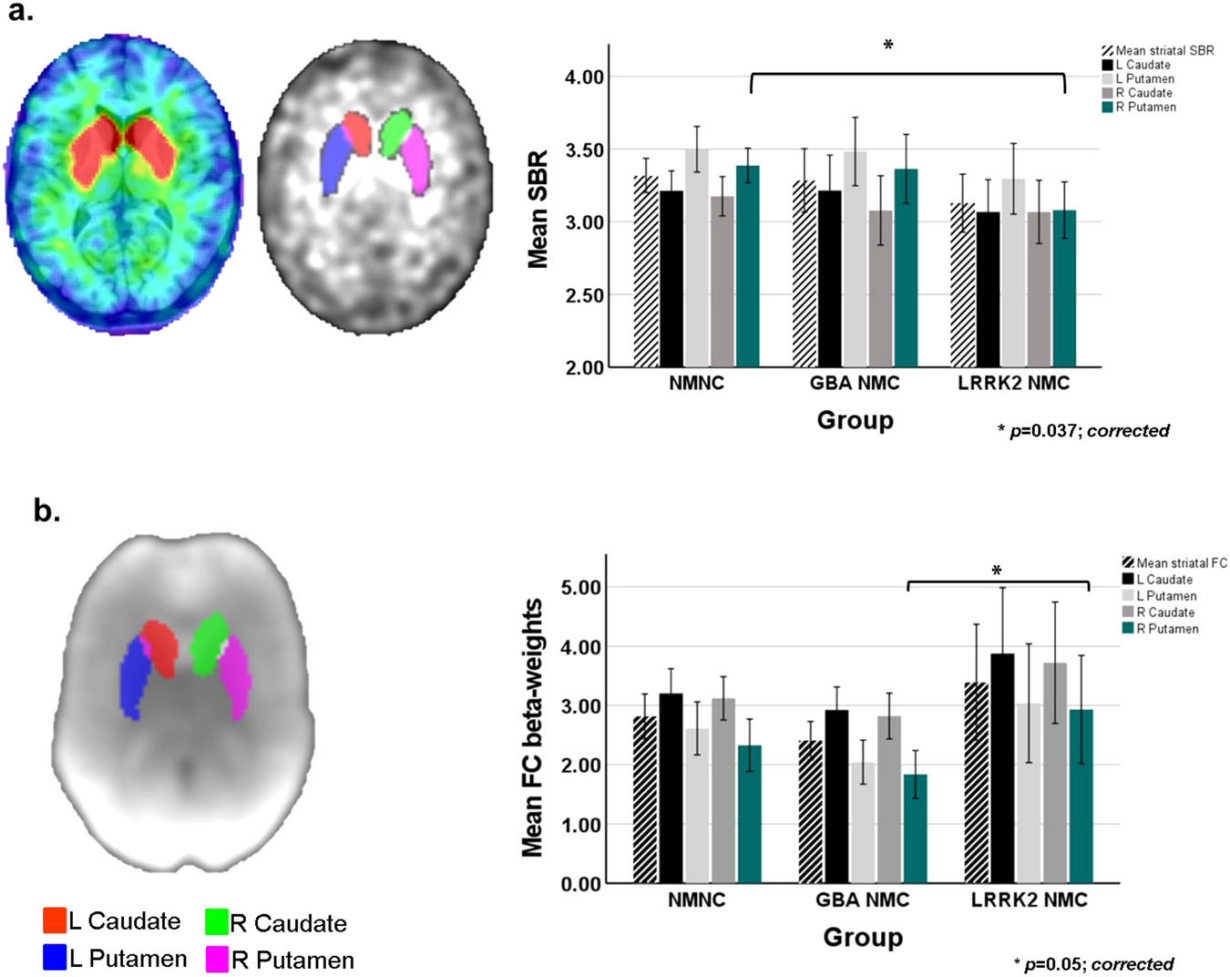
Striatal binding ratios (SBRs) and functional connectivity (FC) levels measured in the three study groups. **a** Illustration of striatal regions of interest (ROIs) superimposed on a DaTscan image (*left panel*). Bar chart demonstrating between-group differences in calculated SBR within the striatal ROIs (*right panel*). **b** Illustration of striatal ROIs superimposed on calculated BGN spatial map (*left panel*). Bar chart demonstrating between-group differences in measured FC levels within the striatal ROIs (*right panel*). Bars represent the calculated group means, standard deviations (SD) are represented by the error bars.

Between groups differences in the measured SBR values are summarized in Table 2a. Significant differences in the right putamen were detected (1 × 3 ANOVA; *F*_(2,75)_, *p* = 0.028). Based on the post-hoc analysis, *LRRK2-*NMCs had significantly lower SBR levels compared with NM-NCs in the right putamen (*p* = 0.037; Bonferroni-corrected). (See Fig. 1a). No significant differences were detected in the left putamen and bi-lateral caudate between the three study groups.

**Table 2:** Measured DaTscan striatal binding ratio (SBR) and rs-fMRI functional connectivity (FC) values within striatal regions of interest (ROIs).

	NM-NC	GBA-NMC	LRRK2-NMC	*p* value	Post-hoc comparisons		
					NM-NC *vs*. GBA-NMC	NM-NC *vs*. LRRK2-NMC	GBA-NMC *vs*. LRRK2-NMC
***a. DaT- SBR***							
**L Caudate**	3.21 ± 0.38	3.21 ± 0.53	3.07 ± 0.46	0.481^a^	–	–	–
**R Caudate**	3.17 ± 0.38	3.08 ± 0.52	3.07 ± 0.45	0.62^a^	–	–	–
**L Putamen**	3.50 ± 0.44	3.48 ± 0.52	3.30 ± 0.50	0.31^a^	–	–	–
**R Putamen**	3.39 ± 0.34	3.36 ± 0.52	3.08 ± 0.40	0.028^a^	–	0.037^b^	–
**Caudate asymmetry [%]**	3.68 ± 3.96	5.44 ± 4.91	5.38 ± 4.63	0.51^a^	–	–	–
**Putamen asymmetry[%]**	6.41 ± 4.88	6.24 ± 5.46	9.35 ± 10.15	0.24^a^	–	–	–
***b. rs-fMRI-FC***							
**L Caudate**	3.12 ± 1.01	2.92 ± 0.85	3.87 ± 2.57	0.15^a^	–	–	–
**R Caudate**	3.12 ± 1.01	2.82 ± 0.85	3.72 ± 2.37	0.14^a^	–	–	–
**L Putamen**	2.61 ± 1.24	2.04 ± 0.82	3.04 ± 2.32	0.12^a^	–	–	–
**R Putamen**	2.33 ± 1.22	1.84 ± 0.88	2.93 ± 2.11	0.05^a^	–	–	0.05^b^

Unless otherwise stated, all values are expressed as mean ± SD.

^a^1 × 3 ANOVA.

^b^Post-hoc comparisons (Bonferroni-corrected).

### Between-groups differences in whole-basal ganglia network (BGN)

**Fig. 2:**
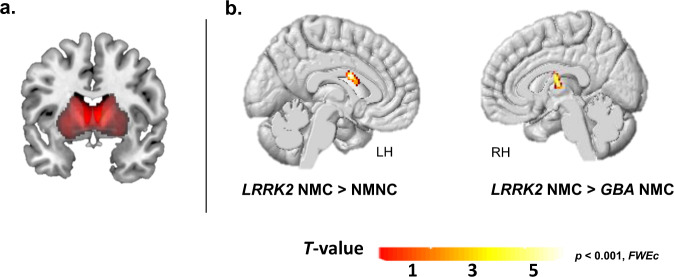
Whole-brain between-group FC differences within the BGN. **a** Spatial distribution illustration of the calculated BGN network. **b** Significantly higher FC levels were observed in the left caudate in the *LRRK2* NMC group compared to the NM-NC group. *LRRK2-*NMCs showed increased FC in the right thalamus compared to *GBA-*NMCs (*p* < 0.001, FWEc).

Compared to NM-NCs, *LRRK2*-NMC group showed significantly higher FC levels in the left caudate (*p* = 0.02, FWEc). Significantly higher FC levels were observed in the right thalamus in *LRRK2-*NMCs compared to *GBA*-NMCs (*p* = 0.01, FWEc) within the BGN as well. (See Fig. 2 & Supplementary Table 1). No significant differences in other brain regions were further detected between the study groups.

### Between-groups differences in striatal rs-fMRI FC levels

Table 2b & Fig. 1b demonstrate the measured intra**-**striatal FC levels in the three study groups. Significant differences in the right putamen were detected (1 × 3 ANOVA; *F*_(2,75)_, *p* = 0.05). Based on the post-hoc comparisons, *LRRK2*-NMC demonstrated significantly higher FC levels in the right putamen compared to *GBA*-NMC (*p* = 0.05; Bonferroni-corrected).

### Association between DaTscan, FC, and likelihood ratio (LR)

**Fig. 3:**
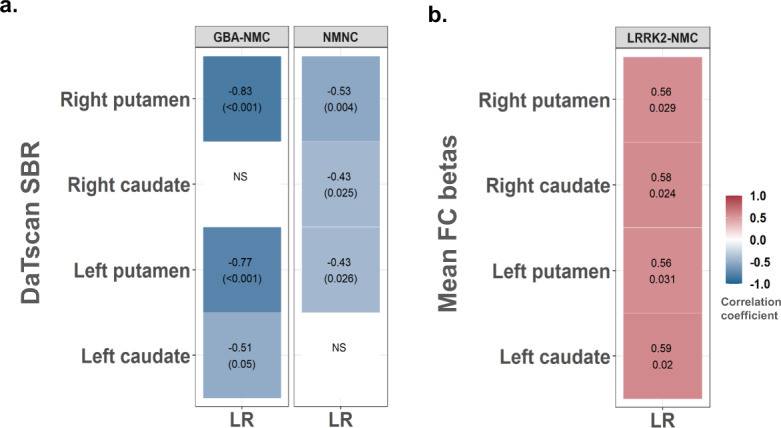
Heat maps demonstrating significant correlation coefficients and *p*-values between LR scores, measured striatal SBRs, and FC levels. **a** Correlations between LR scores and striatal SBR in GBA-NMC and NM-NC groups. **b** Correlations between LR scores and striatal FC levels in the LRRK2-NMC group. The strength and direction of these associations are indicated by the color scale.

Significant results of partial correlations accounting for age and gender between DaTscan metrics, FC, and LR scores in the three study groups are summarized in Fig. 3. In the NM-NC group, LR was negatively associated with SBR in the left putamen (*r* = −0.43, *p* = 0.026), right caudate (*r* = −0.43, *p* = 0.025), and right putamen (*r* = −0.53, *p* = 0.004). In the *GBA*-NMC group, negative associations between LR and SBR in the left caudate (*r* = −0.51, *p* = 0.05), left putamen (*r* = −0.77, *p* < 0.001), and right putamen (*r* = −0.83, *p* < 0.001) were observed (See Fig. 3a). Finally, in the *LRRK2*-NMC group, LR scores correlated positively with measured FC levels in the left caudate (*r* = 0.59, *p* = 0.02), left putamen (*r* = 0.56, *p* = 0.031), right caudate (*r* = 0.58, *p* = 0.024), and right putamen (*r* = 0.56, *p* = 0.029). In this group, no significant associations between the measured striatal FC levels and SBR were detected (Fig. 3b).

### Conversion to definite PD

**Fig. 4:**
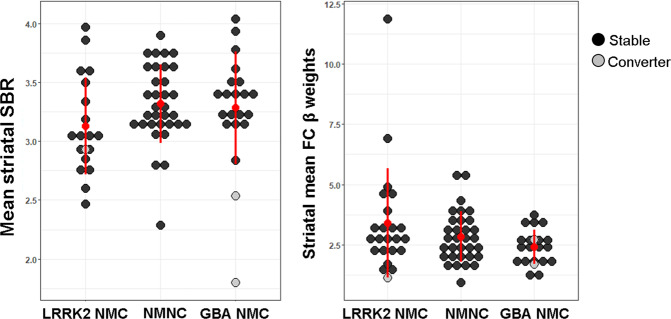
Dot plots of mean striatal SBR (left panel) and FC levels (right panel) in the study groups. Grey dots denote those that converted to definite PD.

Within the first three years since enrollment to the study, three participants (*N* = 2 *GBA*-NMC, and *N* = 1 *LRRK2* NMC) converted to clinically-definite PD. Baseline demographic and clinical characteristics of these participants are demonstrated in Supplementary Table 2. Both *GBA* converters were found to be 3 and 1 SD below the groups’ mean in regards to measured mean striatal SBR respectively. The *LRRK2-*NMC converter was found to be 0.5 SD below the groups’ mean in this measure. In intra-striatal FC level, the *GBA* converters were 2 and 1 SD below the groups’ intra-striatal mean FC level. Similarly, the *LRRK2*-NMC converter was found to be 1 SD below the *LRRK2*-NMC groups’ mean (See Fig. 4 & Supplementary Table 2).

## Discussion

Subtle nigrostriatal dopaminergic pathways dysfunction has been documented in the prodromal phase of PD leading to sub-clinical motor symptoms^17,18^. Neuroimaging plays an important role in investigating changes in these pathways occurring during this phase^2^. This was a single-site study, where we performed a combined DaTscan and rs-fMRI analysis of data collected from *GBA-*NMC and *LRRK2*-NMC to explore the interplay between SBR levels and regional FC in the striatum in individuals at increased risk for PD. Using multi-modality neuroimaging methods, distinct group-specific patterns were observed.

*LRRK2*-NMCs showed significantly reduced striatal SBR in the right putamen compared to the NM-NCs study group, while no significant differences in the measured DaTscan SBRs in the *GBA*-NMCs group compared to NM-NCs were observed. This is in line with recent findings which demonstrated striatal SBR alterations in the pre-clinical stage of the disease^15^. This study further reported a significant SBR increase among *GBA*-NMCs compared to *LRRK2-* NMCs, and NM-NCs^15^. We observed higher left and right putamen SBR among *GBA*-NMCs and *LRRK2*-NMCs, however, these did not reach statistical significance. Discrepancies between the findings of both studies could be attributed to several factors including, the smaller cohort size of the current study, different methodologies used for SBR calculation, and mutation types dissimilarities included within the *GBA*-NMCs group. Nevertheless, this finding highlights the differential degenerative processes affecting striatal dopaminergic neurons among the different genetic groups^18^, possibly suggesting that increased striatal DaT binding in *GBA*-NMCs carriers might reflect compensatory regulatory alterations and DaT downregulation, leading to increased striatal dopaminergic activity^5^.

In rs-fMRI, *LRRK2*-NMCs demonstrated higher intra-network regional FC levels within the left caudate compared to NM-NCs, and within the left thalamus compared to the *GBA*-NMCs group. This is consistent with recent findings demonstrating increased within network connectivity in PD patients with *LRRK2* mutation compared to sporadic and *GBA* patients^19^. However, correction for multiple comparisons on the whole brain level in the BGN between-group comparisons renders this to be less sensitive for detecting subtle changes taking place in the prodromal phase. This is evident based on the results of region-specific ROI analysis demonstrating higher FC levels in the caudate and putamen in the *LRRK2*-NMCs group compared to NM-NC and *GBA-*NMC groups. Opposed to the fact that no significant differences were observed in the measured FC levels within the striatal ROIs between NM-NC and *LRRK2*-NMCs; measured FC levels within the right putamen in this group were found to be significantly higher compared to the *GBA*-NMCs group. It was recently reported that in contrast to *LRRK2*-PD, *GBA*-PD demonstrate FC increases outside the core networks involving cortico-cortical pathways^19^. In a previous study, we demonstrated increased FC and regional activity among *LRRK2-*NMCs as compared to controls while performing motor and cognitive tasks, possibility reflecting compensatory mechanisms^11,20–22^. Such observed patterns of FC alterations were suggested to represent surrogate markers of the more aggressive clinical course of PD in *GBA* mutation carriers^2^.

Increased frontoparietal and fronto-limbic FC, and preserved striatal dopamine uptake were found to be associated with better cognitive performance in PD and patients with Lewy body diseases^23,24^, reflecting patterns of compensatory mechanisms minimizing clinical deficits. However, little information is available on the relationship between DaT and FC patterns in individuals at risk for PD. When investigating the relationship between the MDS probability scores, DaTscan, and rs-fMRI FC parameters, an interesting parity was detected. In both *GBA-*NMCs and NM-NCs groups, higher LR scores were found to correlate negatively with lower DaT binding, while in *LRRK2*-NMC, higher LR scores correlated positively with the measured striatal FC levels. These findings might indicate differential susceptibility of various midbrain structures to PD pathophysiology^25^. Previous studies demonstrated evidence of terminal losses in the pre-symptomatic phase among *LRRK2*-NMCs^26,27^. Based on these findings, we believe that phenotype-specific compensatory mechanisms allow *LRRK2*-NMCs to maintain clinical functions, as the reduction of DaT binding in the right putamen was associated with increased regional FC in the striatum. A recent PET study by Wile et al.^28^ described candidate cellular compensatory mechanisms in both *LRRK2* PD patients and *LRRK2-*NMCs that might underlie the milder [disease] progression time course. In this study, the authors reported elevated serotonin transporter binding in *LRRK2-*NMCs^28^. This might be indicative of increased serotonin neurons density, regulatory changes in synaptic terminal density, or transporter expression. Thus, reactive or compensatory sprouting of serotonergic terminals in the striatum might occur gradually over a prolonged period and possibly contribute to the preservation of dopamine synthesis in *LRRK2-*NMCs^28^.

The laterality effects observed in this study as well as in others are worthwhile mentioning. As the mechanisms generating asymmetry in the prodromal phase of PD are yet not fully clear, two candidate mechanisms are proposed. Firstly, the number of nigral neurons differs from one side to another from birth; hence, the side with a reduced number of neurons reaches the threshold of clinical manifestation earlier^29^. Alternatively, nigral neurons might show heightened vulnerability on one side compared to the other. Therefore, a subtle ongoing degenerative process will result in a marked difference in the vulnerable side^29,30^. Based on our finding, the latter is more plausible, especially in the putamen, where the NM-NCs showed greater SBR compared to the contralateral side. This is further highlighted in the *LRRK2*-NMCs group where SBR in the right putamen was markedly lower than that measured on the left side.

In *GBA*, a multimodality PET study investigating the neurobiology of PD reported no dopamine synthesis reduction in the striatum in asymptomatic *GBA*-NMCs compared with healthy controls^19^. The authors of this study concluded that *GBA* mutations alone were not predictive for dopamine pathology; rather, other genetic and /or environmental factors most likely act in concert with the disease risk^18^. This further corroborates our findings, as well as those reported by Simuni et al.^15^, wherein *GBA*-NMCs, the maintained SBR together with reduced striatal FC based on rs-fMRI may be indicative of the limited neural reserve in these individuals.

The current study reports only cross-sectional data of an ongoing longitudinal study following up *GBA*-NMCs, *LRRK2*-NMCs, and NM-NCs first-degree relatives of PD patients for five years. Within the first three years following enrollment in this study, three NMCs converted to definite PD. Both *GBA-*NMCs converters were found to have higher LR scores as well as low striatal SBR at baseline, while the *LRRK2*-NMCs converter showed to have relatively low LR and mean striatal FC levels. This low LR could be attributed to the fact that DaTscan derived metrics were not included in the LR calculation, leading to LR under-estimation. Additionally, simultaneous DaTscan and MRI imaging acquisition will contribute greatly in canceling out the effects of possible confounders occurring during the elapsed time between both acquisitions, further enhancing the sensitivity for the spatial and temporal relationship between striatal DaT and FC. It is nevertheless worthwhile acknowledging the potential drawbacks of rs-fMRI-based measures. Relying on the BOLD response, FC parameters might be susceptible to several endogenous and exogenous factors that contribute to signal variability^31,32^. These include hormonal effects, blood pressures, body mass index, circadian rhythm, sleep duration, and other vascular parameters^32^. Adherence to standardized acquisition methods would help to minimize these inherit confounders in rs-fMRI measurements.

Future findings from the follow-up data, and in larger sample sizes would allow for further insights regarding the temporal dynamics between striatal DaT, FC patterns, and clinical performance scores in the prodromal phase of the disease.

In summary, the present findings indicate that differential DaT-FC patterns can be observed among the genotypic groups in prodromal PD. These findings warrant further investigations examining the contribution of FC measures to the sensitivity of conversion prediction to definite PD in individuals at high risk.

## Methods

### Study participants

This study is part of the ongoing BEAT-PD observational study taking place at the Tel Aviv Sourasky Medical Center (TASMC). In total, 89 healthy individuals aged ≥ 40 years were included in this study if they had a first-degree relation to PD and did not fulfill the UK PD Brain Bank criteria for PD. Exclusion criteria were as follows: (i) diagnosed neurological or psychiatric disorder, (ii) a malignancy, (iii) HIV, HBV or HCV positive, (iv) claustrophobia and/or other MRI related contraindication. Of those four participants were excluded from MRI scanning due to contraindications (surgical hardware, tattoos/permanent makeup). No significant incidental findings were reported that would lead to excluding further participants from the analysis based on the neuro-radiological assessment of T2-FLAIR images of all participants. All enrolled participants were genotyped for the G2019S-*LRRK2* mutation and 9 common mutations in the *GBA* gene; N370S, R496H, L444P, 84GG, IVS2 + 1G- > A, V394L, 370Rec, and E326K and T369M. This study was approved by the institutional review board (IRB) at the Tel Aviv Sourasky Medical Center. All subjects gave their informed written consent upon participation.

### Clinical assessments

Demographic data including a complete medical history was collected from all participants. Participants underwent comprehensive assessment using the following tools: the MDS-Unified Parkinson’s Disease Rating Scale (MDS-UPDRS)^33^. The Montreal Cognitive Assessment (MOCA) for global cognitive functions^34^, the Beck Depression Inventory (BDI) for mood assessment^35^. The Non-Motor Symptoms Questionnaire (NMSQ)^36^, the Scale of Autonomic Function in PD (SCOPA-AUT)^37^, and the REM sleep Behavior Disorder Questionnaire (RBDq)^38^. Olfaction was tested using the University of Pennsylvania Smell Identification Test (UPSIT)^39^.

### Likelihood ratio (LR) calculation

The above described clinical measures were used for calculating individualized risk for PD as likelihood ratios (LR) based on the 2019 updated MDS research criteria for prodromal PD; excluding PET/SPECT dopaminergic markers^40,41^. Each subject was allocated a ratio between 0 and 100% for risk of prodromal PD. LR above >70% was considered as high-risk for PD.

### Imaging

#### DaTscan acquisition

Before tracer injection, subjects received stable iodine orally (7–10 drops of a saturated solution of potassium iodide) to reduce uptake and radiation exposure of the thyroid gland. 5 mCi (185MBq) of ^123^ioflupane (DaTScan^TM^) was then injected intravenously. Single Photon Emission Tomography (SPECT) acquisition was initiated 3 h post-injection using an Infinia camera (GE Healthcare) with a fan beam collimator. The acquisition protocol was 128 × 128 matrix size and 20 s per frame. Data were reconstructed as following ordered subset expectation maximization with 2 iterations and 10 subsets, attenuation correction with coefficient 0.11, and Butterworth 0.5 Hz filtering with the critical frequency of 0.5 and power 10. No scatter correction was applied.

#### MRI acquisition

MR data were acquired using a 3-T Magnetom Prisma^®^ (Siemens, Erlangen, Germany) MR scanner equipped with a 20 channel phased-array head coil. The MRI protocol included: (1) a high resolution 3D magnetization-prepared rapid gradient echo (MP-RAGE) T1-weighted sequence (TR/TE/TI = 2200/3.22/1100 ms; FA = 9°, voxel size = 1 × 1 × 1 mm^3^, acquisition time: 5:06 min.), (2) axial T2-fluid attenuated inversion recovery (FLAIR) (TR/TE/TI = 8000/117/2370 ms; FA = 150°; voxel size = 0.8 × 0.8 × 5 mm^3^, acquisition time: 2:58 min.), and (3) eyes open 144 volumes resting-state fMRI datasets using single-shot echo-planar imaging (EPI) sequence (TR/TE = 2500/30 ms; FA = 90°, FoV = 220 × 220 mm; 42 axial slices with no gap, voxel size = 2.3 × 2.3 × 3 mm^3^ with no gap, acquisition time = 6:08 min). The DaT-scan and MRI scans were performed on within 6 ± 4 weeks of each other with no specific order.

### DaTscan processing

All imaging analyses were performed using SPM12 (https://www.fil.ion.ucl.ac.uk/spm/software/spm12/). DaTscan data processing included realignment of the DaT-SPECT images to FP-CIT standard template^42^ using rigid-body transformation and spatial normalization. For SBR calculations, intensity was normalized relative to the occipital lobe which served as a reference area^26,27^. The reference area was defined in each subject based on the MNI structural atlas. Finally, atlas-based (Harvard-Oxford atlas, http://cma.mgh.harvard.edu) masks for the caudate and putamen were applied to the subjects’ DaTscan images to extract mean SBR values within striatal regions of interest (ROIs) (See Supplementary Fig. 1). SBR values were calculated as described in Koh et al., 2016^43^. SBR asymmetry indices were calculated as follows:

$${{{\mathrm{Asym}}}}{{{\mathrm{.}}}}\;{{{\mathrm{Index}}}} = \frac{{|\left( {SBR_{left} - SBR_{right}} \right)|}}{{\left( {SBR_{left} + SBR_{right}} \right) \ast 0.5}} \times 100$$

Where the obtained asymmetry index reflects the percentage of deviation from the average ROI SBR of both sides^44^. The calculated SBR values for the striatal ROIs as described above were compared with those obtained from the GE^®^ clinical software using inter-class correlation for absolute agreement. High agreement between measured SBR values in the investigated striatal regions was obtained using both approaches (ICC = 0.7-0.9, *p* < 0.0001) (See Supplementary Fig. 2).

### MRI data processing

For rs-fMRI datasets, the first 4 time frames (~12 s) were removed to allow the MR signal to achieve T1 equilibrium. The following steps were applied: Slice time correction, re-alignment to the first volume image in order to correct for head movement, frames of high inter-frame displacement (>3–3.5 mm) were disregarded from further analysis. Normalization to MNI space using the forward transformation parameters obtained from DARTEL procedure was then carried out, and smoothing using an 8 mm FWHM kernel. The de-noising and filtering of pre-processed rs-fMRI data sets were analyzed using the CONN (v.17) toolbox (McGovern Institute for Brain Research, Massachusetts Institute of Technology, Cambridge; https://web.conn-toolbox.org/), where, linear de-trending, temporal de-spiking, band-pass filtering (0.01 –0.08 Hz) were carried out after regression. The subjects’ six head-motion parameters, white matter (WM), and cerebrospinal fluid (CSF) signal time-course were entered as covariates of no interest in the regression model. Striatal network calculation was performed by CONN toolbox. Here, group-level independent component analysis (ICA) with 20 independent components (IC) approach was used. Individual subject-level functional networks were obtained by GICA1 back-reconstruction^45^. The basal ganglia functional (BGN) network was identified based on dice coefficients (ICC) for best spatial overlap with a default template provided by CONN as well as based on visual inspection for verification of spatial accuracy (IC = 20, see Szewczk-Krolikowski et al.^46^). Additionally, FC levels within striatal regions were assessed in order to match the performed DaTscan analysis. The caudate and putamen masks were applied to the first-level BGN spatial maps of each subject to extract mean FC beta-weights for all subjects.

### Statistical analysis

Statistical analysis was computed for all measures of interest using SPSS^®^, v22 (IBM, Chicago, USA). One-way analysis of variance (1 × 3 ANOVA) was used to probe differences between the study groups in continuous measures (e.g. Age, H&Y, UPDRS-III, UPDRS-total, MOCA, UPSIT, BDI, SCOPA-AUT, RBDQ, NMS, and LR). Between-group gender differences were assessed using chi-square (χ^2^). Whole-brain between-group differences in the BGN were carried out using 1 × 3 ANOVA in SPM. For this comparison, a cut-off threshold of *p* < 0.001 adjusted for cluster size (FWE*c*) based on AlphaSim calculations (cluster size > 70 voxels) was used. 1 × 3 ANOVA was also applied to probe the between-group differences in SBR and FC values measures within the striatal between the study groups. Partial correlations accounting for age and gender (with bootstrapping 1000 samples) were performed to probe the relationship between LR scores and the measured SBR, and FC levels in the striatal ROIs based on DaTscan and rs-fMRI, within the study groups. The significance threshold of *p* < 0.05 Bonferroni-corrected for multiple comparisons was adopted for all conducted comparisons.

### Reporting summary

Further information on research design is available in the Nature Research Reporting Summary linked to this article.

## Supplementary information

Supplemental Materials

Reporting Summary

## Data Availability

De-identified data supporting the findings reported in this work will be available by the corresponding author upon reasonable request by researchers who meet the criteria for access to confidential data (i.e., affiliated to research institution/ hospital).
