# Supplementary material for: Aberrant dopamine transporter and functional connectivity patterns in LRRK2 and GBA mutation carriers
Source: NPJ Parkinsons Dis. 2022 Mar 3;8:20. doi: 10.1038/s41531-022-00285-z (PMC8894349; doi:10.1038/s41531-022-00285-z)
Supplement: Supplementary file 1 — Supplemental Materials [file 41531_2022_285_MOESM1_ESM.pdf]

Supplementary material

Supplementary Table 1: Brain regions showing significant FC differences in 1×3 ANOVA within the investigated striatal functional networks at p < 0.001-*FWEc* cut-off threshold.

**Network**	**Contrast**	**Anatomical region**	**MNI (x,yz)**	**Cluster size (# voxels)**	**Z-value**	**T-value**	***P_{corr}*-value**
Striatal network	LRRK2 NM-MC > NM-NC	L caudate	-8, 2 24	75	4.53	4.88	**0.02**
	LRRK2 NM-MC> GBA-NMC	R thalamus	10 -10 16	70	3.6	3.78	**0.02**

FC = functional connectivity, MNI: Montreal Neurological Institute, R = right, L = left.

Supplementary Table 2: Demographic and clinical charectristics of converters to definite PD at baseline.

	**Converter 1**	**Converter 2**	**Converter 3**
**Age**	65	47	63
**Gender**	Male	Male	Female
**Genetic status**	GBA (N3705)	GBA(IVS-2)	LRRK2
**UPDRS-III**	0	4	0
**UPDRS- Total**	11	12	2
**MOCA**	28	29	25
**LR at baseline**	95.07	90.96	20.78

LR:liklihood ratio, UPDRS: Unified Parkinson's disease rating scale, MOCA: Montreal cognitive assessment. LR: likelihood ratio.

Figures

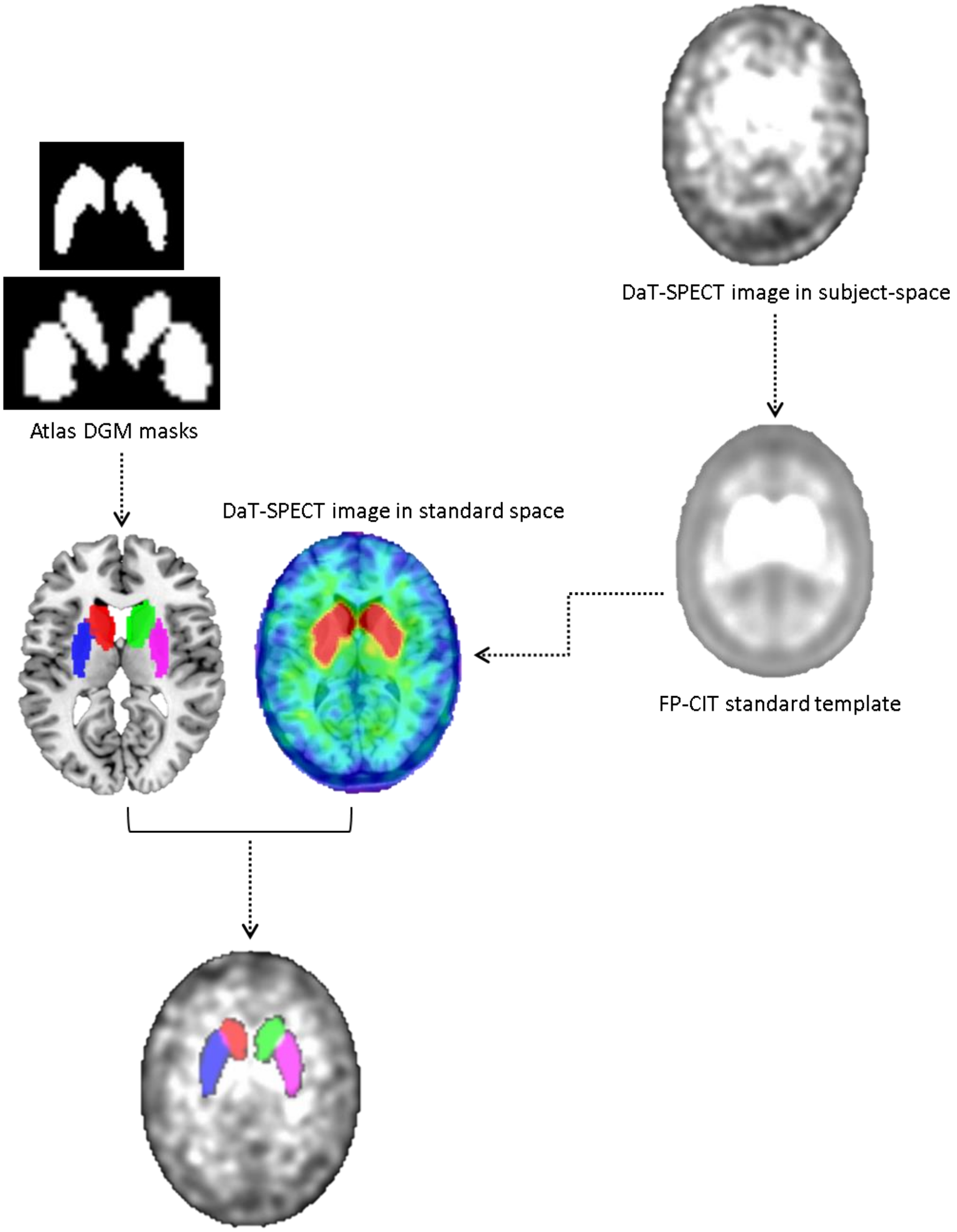

Supplementary Figure 1: **Schematic depiction of the DaTscan processing steps.**

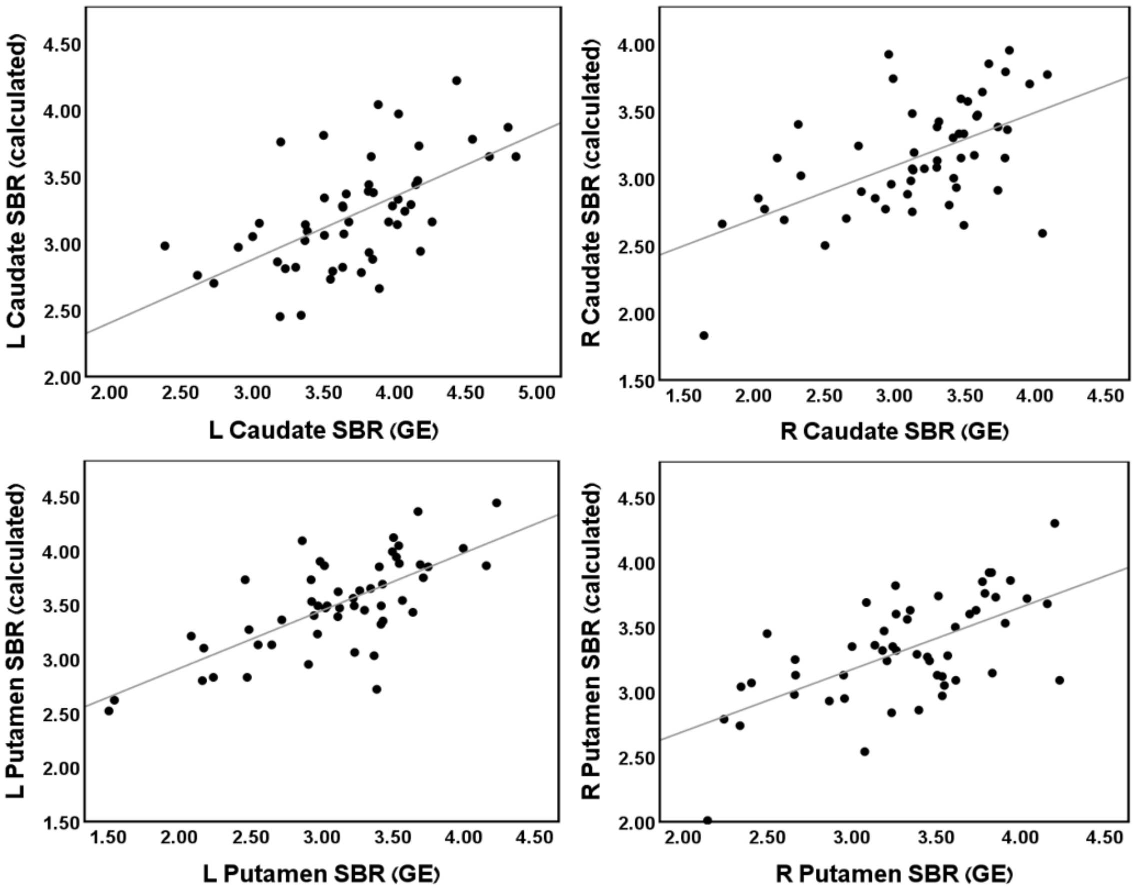

Supplementary Figure 2: **The agreement between the SBR values achieved using two methods.** Scatter plots demonstrate the agreement between the calculated SBR values within the striatal regions of interest (ROIs) obtained from both the clinical imaging software (GE, and the described method in the methods section.
