# Supplementary material for: Aberrant dopamine transporter and functional connectivity patterns in LRRK2 and GBA mutation carriers
Source: NPJ Parkinsons Dis. 2022 Mar 3;8:20. doi: 10.1038/s41531-022-00285-z (PMC8894349; doi:10.1038/s41531-022-00285-z)
Supplement: Supplementary file 2 — Reporting Summary [file 41531_2022_285_MOESM2_ESM.pdf]

nature portfolio

Corresponding author(s): Amgad Droby NPJPARKD-00868

Last updated by author(s): Jan 6, 2022

Reporting Summary

Nature Portfolio wishes to improve the reproducibility of the work that we publish. This form provides structure for consistency and transparency in reporting. For further information on Nature Portfolio policies, see our Editorial Policies and the Editorial Policy Checklist.

Statistics

For all statistical analyses, confirm that the following items are present in the figure legend, table legend, main text, or Methods section.

n/a	Confirmed	
☐	☒	The exact sample size (n) for each experimental group/condition, given as a discrete number and unit of measurement
☐	☒	A statement on whether measurements were taken from distinct samples or whether the same sample was measured repeatedly
☐	☒	The statistical test(s) used AND whether they are one- or two-sided<br>*Only common tests should be described solely by name; describe more complex techniques in the Methods section.*
☐	☒	A description of all covariates tested
☐	☒	A description of any assumptions or corrections, such as tests of normality and adjustment for multiple comparisons
☐	☒	A full description of the statistical parameters including central tendency (e.g. means) or other basic estimates (e.g. regression coefficient) AND variation (e.g. standard deviation) or associated estimates of uncertainty (e.g. confidence intervals)
☐	☒	For null hypothesis testing, the test statistic (e.g. F, t, r) with confidence intervals, effect sizes, degrees of freedom and P value noted<br>*Give P values as exact values whenever suitable.*
☒	☐	For Bayesian analysis, information on the choice of priors and Markov chain Monte Carlo settings
☒	☐	For hierarchical and complex designs, identification of the appropriate level for tests and full reporting of outcomes
☐	☒	Estimates of effect sizes (e.g. Cohen's d, Pearson's r), indicating how they were calculated

Our web collection on statistics for biologists contains articles on many of the points above.

Software and code

Policy information about availability of computer code

Data collection	NA
Data analysis	Statistical analysis was computed for all measures of interest using SPSS®, v22 (IBM, Chicago, USA). Imaging data analysis was performed using statistical parametric mapping (SPM) software operating on Matlab (Mathworks).

For manuscripts utilizing custom algorithms or software that are central to the research but not yet described in published literature, software must be made available to editors and reviewers. We strongly encourage code deposition in a community repository (e.g. GitHub). See the Nature Portfolio guidelines for submitting code & software for further information.

Data

Policy information about availability of data

All manuscripts must include a data availability statement. This statement should provide the following information, where applicable:
- Accession codes, unique identifiers, or web links for publicly available datasets
- A description of any restrictions on data availability
- For clinical datasets or third party data, please ensure that the statement adheres to our policy

The data supporting the findings reported here will be available upon reasonable request by researchers who meet the criteria for access to confidential data.

Field-specific reporting

Please select the one below that is the best fit for your research. If you are not sure, read the appropriate sections before making your selection.

☒ Life sciences ☐ Behavioural & social sciences ☐ Ecological, evolutionary & environmental sciences

For a reference copy of the document with all sections, see nature.com/documents/nr-reporting-summary-flat.pdf

Life sciences study design

All studies must disclose on these points even when the disclosure is negative.

Sample size	85 healthy individuals aged ≥40 years were included in this study if they had a first degree relation to PD and did not fulfill the UK PD Brain Bank criteria for PD.
Data exclusions	Exclusion criteria were as follows: (i) diagnosed neurological or psychiatric disorder, (ii) a malignancy, (iii) HIV, HBV or HCV positive, (iv) MRI related contraindication.
Replication	NA
Randomization	All enrolled participants were genotyped for the G2019S-LRRK2 mutation and 9 common mutations in the GBA gene; N370S, R496H, L444P, 84GG, IVS2+1G->A, V394L, 370Rec, and E326K and T369M. Based on genetic results, the enrolled participants were allocated to one of 3 study groups.
Blinding	Investigators were blinded to genetic status of the participants.

Reporting for specific materials, systems and methods

We require information from authors about some types of materials, experimental systems and methods used in many studies. Here, indicate whether each material, system or method listed is relevant to your study. If you are not sure if a list item applies to your research, read the appropriate section before selecting a response.

Materials & experimental systems

n/a	Involved in the study	
☒	☐	Antibodies
☒	☐	Eukaryotic cell lines
☒	☐	Palaeontology and archaeology
☒	☐	Animals and other organisms
☐	☒	Human research participants
☐	☒	Clinical data
☒	☐	Dual use research of concern

Methods

n/a	Involved in the study	
☒	☐	ChIP-seq
☒	☐	Flow cytometry
☐	☒	MRI-based neuroimaging

Human research participants

Policy information about studies involving human research participants

Population characteristics	Age and gender were entered as covariates in the performed statistical analyses.
Recruitment	individuals aged ≥40 years were included in this study if they had a first degree relation to PD and did not fulfill the UK PD Brain Bank criteria for PD.
Ethics oversight	This study was approved by the institutional review board (IRB) at the Tel Aviv Sourasky Medical Center. All subjects gave their informed written consent prior to participation.

Note that full information on the approval of the study protocol must also be provided in the manuscript.

Clinical data

Policy information about clinical studies

All manuscripts should comply with the ICMJE guidelines for publication of clinical research and a completed CONSORT checklist must be included with all submissions.

Clinical trial registration	NA
Study protocol	NA

Data collection	NA
Outcomes	NA

Magnetic resonance imaging

Experimental design

Design type	Resting state
Design specifications	eyes open 144 volumes resting-state fMRI datasets using single-shot echo-planar imaging (EPI) , 6:08 minutes.
Behavioral performance measures	NA

Acquisition

Imaging type(s)	Structural, Functional
Field strength	3 Tesla
Sequence & imaging parameters	(1) a high resolution 3D magnetization-prepared rapid gradient echo (MP-RAGE) T1-weighted sequence (TR/TE/TI = 2200/3.22/1100 ms; FA = 9°, voxel size = 1 × 1 × 1 mm3 , acquisition time: 5:06 min.), (2) axial T2-fluid attenuated inversion recovery (FLAIR) (TR/TE/TI = 8000/117/2370 ms; FA = 150°; voxel size = 0.8 × 0.8 × 5 mm3 , acquisition time: 2:58 min.), and (3) eyes open 144 volumes resting-state fMRI datasets using single-shot echo-planar imaging (EPI) sequence (TR/TE = 2500/30 ms; FA = 90°, FoV= 220 × 220 mm; 42 axial slices with no gap, voxel size = 2.3 × 2.3 × 3 mm3 with no gap, acquisition time = 6:08 min).
Area of acquisition	Whole brain

Diffusion MRI ☐ Used ☒ Not used

Preprocessing

Preprocessing software	All imaging analyses were performed using SPM12 (https://www.fil.ion.ucl.ac.uk/spm/software/spm12/).
Normalization	Normalization to MNI152 space using the forward transformation parameters obtained from DARTEL procedure was then carried out, and smoothing using an 8 mm FWHM kernel.
Normalization template	MNI152
Noise and artifact removal	For rs-fMRI datasets, the first 4 time frames (~12sec) were removed to allow the MR signal to achieve T1 equilibrium. The following steps were applied: Slice time correction, re-alignment to the first volume image in order to correct for head movement, frames of high inter-frame displacement (> 3-3.5mm) where disregarded from further analysis. For rs-fMRI datasets, the first 4 time frames (~12sec) were removed to allow the MR signal to achieve T1 equilibrium. The following steps were applied: Slice time correction, re-alignment to the first volume image in order to correct for head movement, frames of high inter-frame displacement (> 3-3.5mm) where disregarded from further analysis. Normalization to MNI space using the forward transformation parameters obtained from DARTEL procedure was then carried out, and smoothing using an 8 mm FWHM kernel. The de-noising and filtering of pre-processed rs-fMRI data sets were analysed using the CONN (v.17) toolbox (McGovern Institute for Brain Research, Massachusetts Institute of Technology, Cambridge; https://web.conn-toolbox.org/), where, linear de-trending, temporal de-spiking, band-pass filtering (0.01 Hz – 0.08Hz) were carried out after regression. The subjects' six head-motion parameters, white matter (WM), and cerebrospinal fluid (CSF) signal time-course were entered as covariates of no interest in the regression model.
Volume censoring	CSF and WM signal time series extracted based on tissue probability maps achieved from T1-image segmentation in SPM.

Statistical modeling & inference

Model type and settings	One-way analysis of variance (1×3 ANOVA) was used to probe differences between the study groups in continuous measures.
Effect(s) tested	Whole-brain between-group differences in the BGN were carried out using 1×3 ANOVA. ANOVA was also applied to probe the between-group differences in FC values measures within the striatal between the study groups

Specify type of analysis: ☐ Whole brain ☐ ROI-based ☒ Both

Anatomical location(s)	Basal ganglia (Left+ Right caudate nucleus and putamen).
Statistic type for inference (See Eklund et al. 2016)	Voxel-wise
Correction	A cut-off threshold of p<0.001 adjusted for cluster size (FWEc) based on alphaSim calculations (cluster size > 70 voxels)

Models & analysis

n/a	Involved in the study
☐	☒ Functional and/or effective connectivity
☒	☐ Graph analysis
☒	☐ Multivariate modeling or predictive analysis

Functional and/or effective connectivity	Group-level independent component analysis (ICA) with 20 independent components (IC) approach was used. Individual subject-level functional networks were obtained by GICA1 back-reconstruction.44 The basal ganglia functional (BGN) network was identified based on dice coefficients (ICC) for best spatial overlap with a default template provided by CONN as well as based on visual inspection for verification of spatial accuracy (IC=20, see 45).
